# Geographic variation in gender gap among older adults in India and China: Application of a new ageing measure

**DOI:** 10.1093/geroni/igab046.3152

**Published:** 2021-12-17

**Authors:** Arun Balachandran, Feinian Chen

**Affiliations:** 1 University of Maryland College Park, Hyattsville, Maryland, United States; 2 University of Maryland College Park, University of Maryland, Maryland, United States

## Abstract

A continuous rise in the life expectancy of females above that of the males among older adults in India and China may give an impression that the gender gap in health is decreasing. However, given the systemic bias against females in these countries across multiple facets, and the diversity across provinces, a fuller understanding of gender gap calls for (a) understanding the gender gap in multiple dimensions of health, and (b) understanding the variations across provinces. We estimate a multi-dimensional old-age threshold (MOAT) across provinces in India and China, that specifies different old-age thresholds for female and male populations after simultaneously accommodating for multiple dimensions related to their health. These aspects of health include remaining life expectancy, intellectual and functional health. We estimate the gender gap across provinces in these countries by differencing the MOAT of males against that of females. In addition, we also illustrate the gender gap across individual dimensions of health. Our results show that females in almost all the provinces of India and China have a lower MOAT than their male counterparts, showing an earlier advent of ‘old-age’ among females compared to males. The estimates based on remaining life expectancy shows gender gap in favor of females, but the estimates of multi-dimensional gender gap are higher and biased against females. A huge variation is seen across provinces, with Karnataka and Hubei showing lower levels of gender gap and Rajasthan and Yunnan showing higher gender gaps in India in China respectively.

